# Highly Efficient Extraction Procedures Based on Natural Deep Eutectic Solvents or Ionic Liquids for Determination of 20-Hydroxyecdysone in Spinach

**DOI:** 10.3390/molecules25204736

**Published:** 2020-10-15

**Authors:** Sylwia Bajkacz, Kornelia Rusin, Anna Wolny, Jakub Adamek, Karol Erfurt, Anna Chrobok

**Affiliations:** 1 Department of Inorganic Chemistry, Analytical Chemistry and Electrochemistry, Faculty of Chemistry, Silesian University of Technology, B. Krzywoustego 6, 44-100 Gliwice, Poland; korneliasliwka@op.pl; 2 Biotechnology Center of Silesian University of Technology, Krzywoustego 8, 44-100 Gliwice, Poland; jakub.adamek@polsl.pl; 3 Department of Organic Chemical Technology and Petrochemistry, Faculty of Chemistry, Silesian University of Technology, Krzywoustego 4, 44-100 Gliwice, Poland; ancziixx@gmail.com (A.W.); karol.erfurt@polsl.pl (K.E.); Anna.Chrobok@polsl.pl (A.C.); 4 Department of Organic and Bioorganic Chemistry and Biotechnology, Faculty of Chemistry, Silesian University of Technology, Krzywoustego 4, 44-100 Gliwice, Poland

**Keywords:** natural deep eutectic solvent, ionic liquid, ecdysone, spinach, natural products

## Abstract

A novel, efficient extraction procedure based on natural deep eutectic solvents (NADES) and ionic liquids (ILs) for determination of 20-hydroxyecdysone (20-E) in spinach has been developed. NADES, the first green extraction agent, with different hydrogen bond donors and acceptors are screened in order to determine extraction efficiencies. NADES consisting of lactic acid and levulinic acid at a molar ratio of 1:1 exhibits the highest yields. ILs, the second green extraction agent, with various cations and anions are also investigated, where [TEA] [OAc]·AcOH, χ_AcOH_ = 0.75 displays the highest recovery. Moreover, NADES-SLE and IL-SLE (SLE, solid-liquid extraction) parameters are investigated. Using the obtained optimized method, the recoveries of the target compound in spinach are above 93% and 88% for NADES-SLE and IL-SLE procedure, respectively. The methods display good linearity within the range of 0.5–30 μg/g and LODs of 0.17 µg/g. The proposed NADES-SLE-UHPLC-UV and IL-SLE-UHPLC-UV procedures can be applied to the analysis of 20-E in real spinach samples, making it a potentially promising technique for food matrix. The main advantage of this study is the superior efficiency of the new, green extraction solvents, which results in a significant reduction of extraction time and solvents as compared to those in the literature.

## 1. Introduction

Ecdysteroids are steroid hormones that were first discovered in silkworm larvae in 1954. In the 1960s, researchers showed that they could also be produced by plants. Ecdysones belong to polar ecdysteroids, which are generated by plants to protect against insects. Moreover, biologically active phytoecdysones affect many physiological functions of mammals and display a wide range of pharmacological properties, such as protein synthesis and anti-cancer [1].

Ecdysones have been discovered in 150 above-ground plant families, but only a few of them have high concentrations (about 1%), these include the Chenopodiaceae family, e.g., spinach (50 µg/g dry mass) [2]. The onset of ecdysteroid production in spinach requires the appropriate ontogenetic development within the plant, which is related to leaf development. Importantly, the content of these compounds changes depending on season or development phase, as well as geographical location, natural environment, and plant variety [3]. According to reports, the highest concentrations occur during flowering.

**Figure 1:**
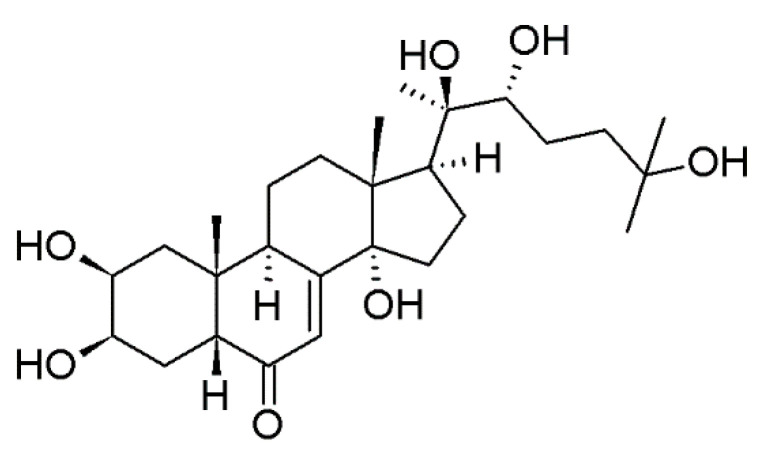
Chemical structure of 20-hydroxyecdysone (ecdysterone, 20-E).

20-hydroxyecdysone (20-E) is the most common ecdysone in the plant and mammalian world (Figure 1). In animals, it acts as a molting hormone, whereas in plants it protects against pest attack. Additionally, plants greater amounts (up to 3% dry mass) compared to animals (up to 0.025% dry mass). Various studies are examined the pharmacological properties of this compound. 20-E stimulates the formation of antibodies, reduces the amount of cholesterol, and is responsible for the anabolic and hypoglycemic effect [4].

Many analytical methods for the study of ecdysone in plants have been established, including thin layer chromatography (TLC) [5,6,7], high performance liquid chromatography [8,9,10], ultra-high performance liquid chromatography [11,12] with diode array detector (HPLC-DAD or UHPLC-DAD) and liquid chromatography-mass spectrometry or (LC-MS) [13,14].

Sample extraction and clean-up are normally required prior to chromatographic analysis due to the matrix effect. Reports have shown that methanol [9,10,14], ethanol [12,13], and ethyl acetate [8] are crucial solvents for the extraction of ecdysones from plants. However, the majority of known extraction methods have drawbacks, such as time consuming procedures, large consumption of toxic solvents, costly equipment, and complicated sample preparation procedures. Therefore, an economical and environmentally friendly extraction method of phytoecdysones is highly desirable.

The development of green extraction agents as a substitute for organic solvents is mainly focused on natural deep eutectic solvents (NADESs) [15] and ionic liquids (ILs) called “green solvents” [16]. Additionally, NADESs are a modern type of green solvents with comparable properties to ILs and can possibly replace conventional solvents and ILs. NADESs are merely generated by mixing two or more naturally obtaining, inexpensive, and biodegradable constituents that are susceptible to self-association, mostly via hydrogen bond interactions, to form a eutectic mixture that exhibits melting points significantly lower than individual compounds [15,16].

Compared to standard organic solvents, NADESs and ILs display unusual properties, such as thermal and chemical stability, non-toxicity, and non-flammability. Other IL properties—including density, viscosity, polarity, and hydrophobicity—can be ‘designed’ by matching a cation and anion entering the IL structure [17].

ILs can be applied to the extraction of various compounds from plants, such as galantamine [18], indolylbutyric acid [19], caffeoylquinic acids [20], glaucyna [21], rutin, and quercetin [22]. In all cases, extraction is supported by sonication or mixing to enhance efficiency. NADESs have also been used as solvents to extract a wide range of non-polar and polar bioactive compounds from plant materials in combination with ultrasound-assisted extraction (UAE) [23,24,25] and microwave-assisted extraction (MAE) [26,27]. However, the extraction of ecdysones from spinach by ILs and NADESs has not yet been reported.

The main goals of this study were to develop an IL and NADES-based extraction procedure for isolation of 20-hydroxyecdysone from spinach. In the experiment, the key factors affecting the extraction efficiency were screened and optimized. Finally, the optimized extraction and determination conditions (NADES-SLE-UHPLC-UV and IL-SLE-UHPLC-UV) were used to determine the 20-E in spinach samples.

Utilization of NADES and ILs as a substitute for a standard organic solvents ultimately generates a more ecological, cheaper, faster, and safer method.

## 2. Results and Discussion

### 2.1. Development of Chromatographic Method

The chromatographic conditions were tested to obtain separation of analyte from matrix components in short time. Different types of columns (Zorbax RRHD SB-C18 (50 × 2.1 mm, 1.8 μm), Zorbax 300SB-C18 (150 × 4.6 mm; 2.7 µm), Hypersil GOLD™ (100 × 2.1 mm, 1.9 μm), mobile phases (acetonitrile–water, methanol–water), types of acid (trifluoroacetic acid, formic acid, and acetic acid), concentrations of additive (0.05%, 0.1%, and 0.2%), column temperatures (20, 30, and 40 °C), and flow rates (0.4, 0.6, 0.8, and 1.0 mL/min) were tested.

It was found that the resolution and symmetry of the peaks decreased when using the Zorbax RRHD SB-C18 and Hypersil GOLD™ columns when compared to the Zorbax 300SB-C18 column. This column, due to the unique, superficially porous particle and 2.7-μm particle size provides high resolution of tested analyte and other sample components. In addition, this column enabled to obtain robust, symmetrical peaks, and higher sensitivity of the method compared to other columns. Therefore, the Zorbax 300SB-C18 column was selected as the analytical column. The analyte retention time was shorter using acetonitrile-water compared to methanol-water, so acetonitrile-water was chosen as the mobile phase. The results showed that the addition of 0.05% trifluoroacetic acid into the water phase worked best, especially in terms of a good symmetric peak. Then several gradient programs were compared and the percentage of acetonitrile was checked to obtain a proper separation of 20-E from the interference from spinach samples. The column temperature was kept at 30 °C and the flow rate was 1 mL/min. The total chromatography analysis run time per sample using the developed method was 8.0 min. To detect 20-E with high sensitivity, the compound was monitored at its maximum absorption wavelength (λ = 242 nm).

### 2.2. Selection of NADES Extraction Parameters

#### 2.2.1. Effect of NADES Composition

**Figure 2:**
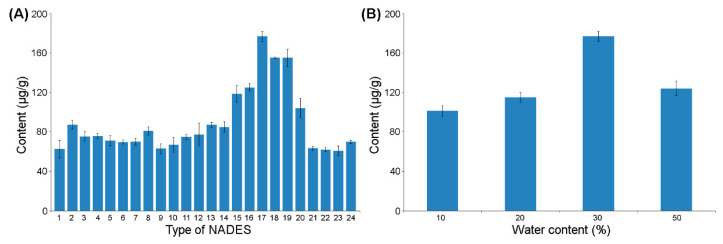
Effect of NADES type (**A**) and water content (**B**) on 20-E extraction efficiency from spinach.

Twenty four different NADES compositions were tested to determine the most suitable NADES for 20-E extraction from *Spinacia oleracea* L (Figure 2A). The obtained solvents had high viscosity, hence, 30% (*w*/*w*) water in NADES solution was used for the extraction.

Each NADES contained two or three compounds but at least one of the components is a carboxylic acid (lactic, malic, levulinic, tartaric, citric, or pyruvic acids). The pK_a_ values of acids were in the range of 2.65 to 4.78. By comparing solvents, it showed that stronger acids form a eutectic mixture, which was more effective for extraction of selected ecdysone.

The most effective extraction systems were obtained when lactic acid and levulinic acid was used as NADES components (especially NADES 17; lactic acid:levulinic acid; 1:1). It seems that the specific supramolecular structure of NADES 17, based mainly on the HBD-HBA interaction, enables also the very efficient formation of hydrogen bonds with the targeted compound, which has a great positive impact on the 20-E extraction process. Moreover, NADESs 17 (lactic acid:levulinic acid; 1:1) was clear and transparent with relatively low viscosities at room temperature.

The obtained results indicate also that polarity and viscosity (which can be modulated in some range by using water, see the next section) play an important role in the NADES extraction.

#### 2.2.2. Effect of Water Content in NADES

**Figure 3:**
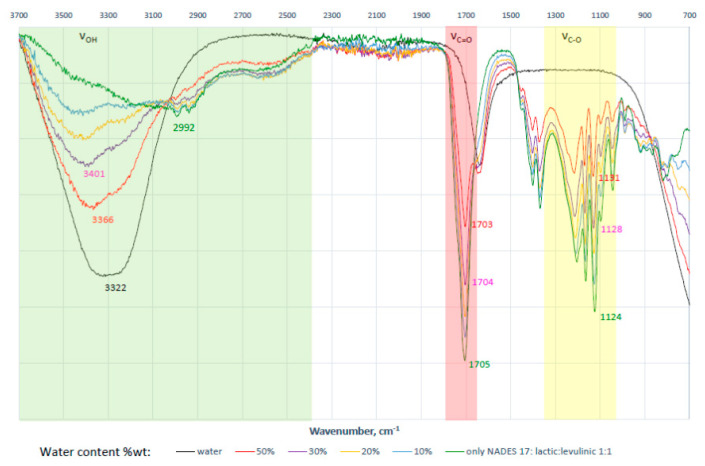
Changes in the supramolecular structure of NADES-17 induced by water content (based on FT-IR spectra).

Extractions were carried out at different water contents in NADES (from 10% to 50%, *v*/*v*) (Figure 2B). The addition of water to the natural solvent can result in a decrease of extractant viscosity and, consequently, promote more efficient mass-transfer rate of ecdysone to improve extraction efficiencies. At 10% and 20% water content, the solvent’s viscosity was too high and hindered thorough mixing of the sample with the solvent. In the case of 50% water content, the extraction efficiency decreased due to the additional water not only changing the physical properties of NADES (e.g., viscosity) but also modifying its supramolecular structure and polarity (Figure 3). Therefore, a large amount of water (>30 wt%) impedes or decreases hydrogen bonding interactions between NADES and target ecdysone.

The optimal water content was determined as 30% because, at this level, water reduced solvent viscosity, while not affecting the interaction solvent-analyte. In general, except for the above advantages of water addition, water could be also added to adjust the polarity of the solution.

#### 2.2.3. Effect of Sample Weight

The impact of the sample weight (50, 100, 150 mg) on efficiency was analyzed (Figure S1A). When NADES volume in relation to the sample was large, its analyte extraction capacity was high and required less extraction time. However, when insufficient amount of solvent was used, the extraction became sluggish and decreased efficiency over time. At 50 mg and 100 mg sample weight, the obtained concentrations were comparable. Hence, extraction efficiency was mainly dominated by the dissolving capability of the solvents. For example, some compounds containing higher molecular weight—such as cellulose, lignin, etc.—may also hamper extraction when excess plant powders were added to NADESs.

Guided by the economics of the method, conditions were chosen in which the consumption of the solvent was as small as possible. The poor extraction effect observed for 150 mg sample may result from insufficient mixing of the sample with NADES, which may promote inferior mass transfer. Therefore, 100 mg of sample was selected for further analysis.

#### 2.2.4. Effect of Extraction Time

The influence of shaking time was examined by varying the extraction time (10, 20, and 40 min). The optimal extraction time was determined as 20 min. As shown in Figure S1B, signal values were elevated as the vortex time increased from 10 to 20 min, however, insignificant difference were observed at 20 and 40 min. However, reduced shaking time considerably increased the overall procedure-time for 20-E determination. Moreover, long-term extraction using NADES was not suitable for bioactive compounds as it could alter the chemical structures of the target compounds. Fortunately, our method had comparatively short extraction time, which added stability to NADESs for effective extraction.

### 2.3. Selection of the IL Extraction Parameters

#### 2.3.1. Effect of Extraction Solvents

**Figure 4:**
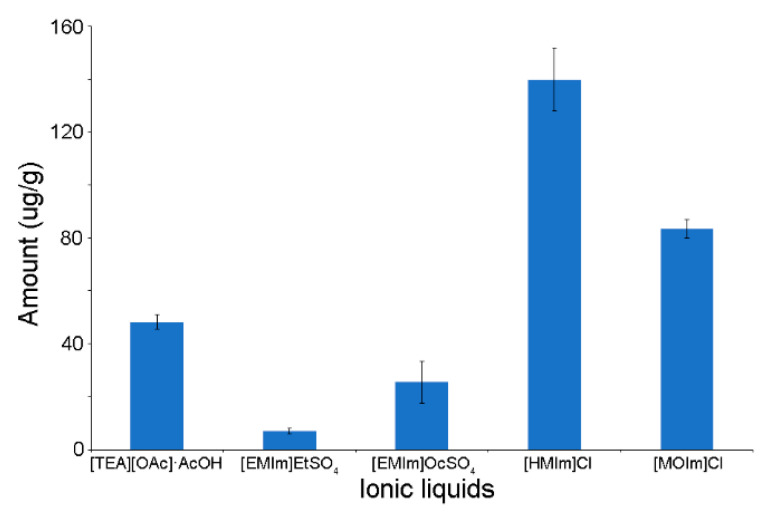
Effect of IL type on 20-E extraction efficiency from spinach.

9 ILs were investigated, and the results showed solvent type had a decisive influence on 20-E extraction efficiency (Figure 4). Among all ILs tested only five displayed effective extraction of 20-E from spinach. However, only [TEA] [OAc]·AcOH, χ_AcOH_ = 0.75 exhibited selective extraction of 20-E, and compared to other organic solvents, possessed optimal extraction efficiency. The obtained peak area values for ionic liquids with chloride anion ([HMIm] Cl and [MOIm] Cl) were overestimated due to the influence of matrix components eluting at the same time as the analyte. In the chromatogram for the extract obtained after using [TEA] [OAc] AcOH, the peaks are already well separated.

Effective and selective extraction of 20-E using [TEA] [OAc]·AcOH was due to the formation of a large number of hydrogen bonds between particles, originating from the structure of 20-E and extractant. Analysis of the selected IL showed that hydrogen bonds were present in the anion. Efficiency of extraction of 20-E increases/decreases with electrophilicity/nucleophilicity of the IL anion. By comparing the alkalinity of ILs anions based on Gutmann Donor Numbers (Table S1), the higher donor number value, the greater the anion alkalinity being related to the ILS. The cation effect was not noticeable. It could only increase of alkalinity of tested ionic liquids.

#### 2.3.2. Effect of IL Dilution, pH, Solid/Liquid Ratio, and Extraction Time

Optimization of IL dilution, pH, sample mass and extraction time was achieved by changing each parameter separately. The comparison of 20-E content after extraction (Figure S2A) showed that dilution of IL had a positive effect on extraction efficiency, in which dilution with water gave an eight-fold enhancement compare to undiluted solvent. The dilution of IL decreased solvent viscosity, which promoted better mix of components and increased polarity of extractant. 20-E is a polar compound, hence, extraction with diluted [TEA] [OAc]·AcOH was more effective. Additionally, the extent of solvent dilution had little influence on the result. The results revealed that optimal IL/water ratio was 2:1 (*v*/*v*), producing the largest 20-E content in the extract. According to results displayed in Figure S2B, pH 7 was deemed optimal for extraction.

The sample mass was tested by weighing various amount of spinach and extraction using 1 mL of IL. The best result was obtained weighing 0.25 g of sample and extraction by 1 mL of solvent (Figure S2C). A similar value of the 20-E content gave extraction of 0.35 g spinach, but was no need to use a greater amount of sample at the same extraction efficiency.

As shown in Figure S2D, longer extraction time was beneficial to extraction efficiency, in which 120 min was determined as the best extraction time.

Based on the experimental date, the optimum IL-SLE extraction conditions were as follows: [TEA] [OAc]·AcOH/water ratio, 2:1 (*v*/*v*); IL pH, 7; solid/liquid ratio, 0.25/1 (g/mL); extraction time, 120 min.

### 2.4. Comparison of NADES and IL Extraction with Other Solvents

The developed procedure with NADES and IL (NADES-SLE and IL-SLE) was compared with conventional extraction techniques based on shaking the sample with water, methanol, acetone, ethyl acetate and methanol:ethanol:water mixture (1:1:1; *v*/*v*/*v*). The proposed NADES and IL approach improved the extraction efficiency of the target analyte compared to SLE of other solvents (Figure S3). The worst results were obtained with less polar solvents, i.e., ethyl acetate and acetone, and were enhanced slightly using methanol or water. The methanol:ethanol:water mixture (1:1:1; *v*/*v*/*v*) gave the best extraction results among conventional solvents, however, the obtained extraction yields were approx. 20% lower those of NADES and IL.

### 2.5. Analytical Figures of Merit

Under optimal conditions, performances of the developed methods were evaluated by considering linear dynamic range (LDR), precision and accuracy (within-day and between-day), limit of detection (LOD), limit of quantification (LOQ), and recoveries (R). The figures of merit of the developed procedures are presented in Table 1. The linear range of the method was obtained in the range of 0.5–30 µg/g with a correlation coefficient of 0.9982 for NADES-SLE-UHPLC-UV, and 0.9994 for IL-SLE-UHPLC-UV. The within-day and between-day precisions of the developed method via analysis of six replicates at concentration levels of 1, 10, and 25 µg/g were below 7% for NADES-SLE-UHPLC-UV and 8% for IL-SLE-UHPLC-UV. In order to verify the reliability of the method, the recovery experiments were carried out on blank spinach samples with the spiked levels of 1 µg/g, 10 µg/g, 25 µg/g, respectively. 20-E recoveries were in the range of 86.2–88.8% with RSDs from 1.8% to 6.5% for IL-SLE-UHPLC-UV and 88.1–93.4% with RSDs from 2.5% to 8.7% for NADES-SLE-UHPLC-UV.

### 2.6. Application of the Developed Method to Real Samples

**Figure 5:**
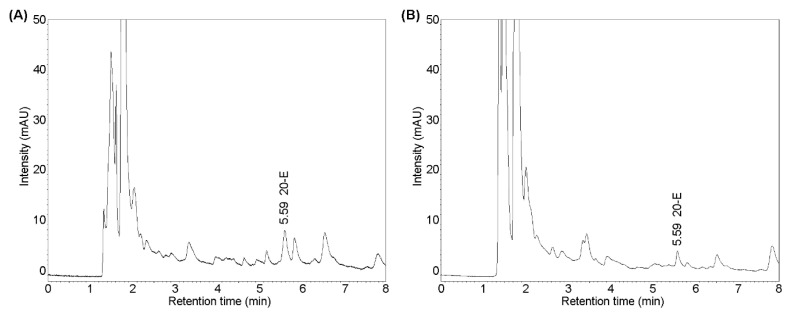
Representative chromatograms obtained for extract of *Spinacia oleracea* L using the proposed NADES-SLE-UHPLC-UV method (spinach leaves (**A**) and spinach stalks (**B**)).

The present NADES-SLE and IL-SLE procedures were used for the determination of 20-E in several spinach samples including fresh spinach, frozen spinach, spinach leaves, spinach stalks, and spinach seeds. Each sample was prepared in triplicate under optimal conditions and then quantified under suitable UHPLC-UV method. The obtained results are shown in Table 2. The determined 20-E contents differed depending on the type of spinach, with Matador spinach showing the highest 20-E content. The results also show that the largest amount of ecdysone was found in young spinach leaves, much less in the stems, and spinach seeds having the lowest content. The obtained results were in agreement with those described in the literature. Additionally, 20-E content was almost 2-fold higher in fresh spinach than in frozen products, which was probably related to its processing stages. Figure 5 illustrates typical chromatograms of fresh spinach samples after NADES-SLE, which confirmed the high clean-up ability of NADES in the extraction of 20-E from spinach samples. The results demonstrated that the established method was suitable for 20-E analysis in different kinds of spinach samples.

### 2.7. Comparison of Proposed NADES-SLE-UHPLC-UV and IL-SLE-UHPLC-UV Methods with Other Reported Methods

The extraction efficiency of 20-E from spinach samples using the proposed method was compared with that of reported procedures (Table 3).

The proposed method advantages include simplicity, high efficiency, rapidity, and high recovery, as well as very low consumption of organic solvents compared to UAE [30] and SLE [12,31] resulting in reduced wastes. The selectivity of NADES-SLE-UHPLC-UV and IL-SLE-UHPLC-UV methods was considerably greater than the literature methods. Moreover, the total analysis time was less in comparison with the developed procedures based on HPLC-UV [30]. The recovery of the proposed method was similar or better than reported methods even when using longer extraction procedures [12,28,29,30,31].

## 3. Experimental

### 3.1. Chemicals and Materials

Choline chloride, acetylcholine chloride, citric acid, levulinic acid, DL-malic acid, l(+)-tartaric acid, l-lactic acid, and pyruvic acid were purchased from Alfa Aesar (Lancashire, United Kingdom). 1-Butyl-3-methylimidazolium bis (trifluoromethylsulfonyl) imide, 1-butyl-3-methylimidazolium methyl sulfate, 1-butyl-3-methylimidazolium tetrachloroaluminate, and 1-ethyl-3-methylimidazolium octyl sulfate, 1-ethyl-3-methylimidazolium trifluoromethane-sulfonate, 1-ethyl-3-methylimidazolium ethyl sulfate, 1-hexyl-3-methylimidazolium chloride, and 1-methyl-3-octylimidazolium chloride, were purchased from Sigma-Aldrich (St. Louis, MO, USA). Analytical standard 20-hydroxyecdysone was purchased from Sigma-Aldrich (St. Louis, MO, USA). HPLC grade acetonitrile (ACN) and trifluoroacetic acid (TFA) were purchased from Merck (Darmstadt, Germany). Double distilled water was prepared using a Milli-Q water purification system. All other chemicals were at least of analytical grade.

Fresh and frozen *Spinacia oleracea* L. and spinach seeds were purchased from the local market in Poland. Plants were dried at 50 °C, ground using a laboratory blender and were stored in glass bottles under room conditions for further analysis.

Centrifuges (model Mini G) from IKA (Wilmington, NC, USA), ultrasonic bath from Polsonic (Warsaw, Poland), and shaker (model Vibramax 100) from Heidolph (Schwabach, Germany) were used for the experiments, respectively.

Infrared (IR) spectra of lactic acid, levulinic acid, their mixture (NADES 17, molar ratio of 1:1), and eutectic mixture with different water content (NADES 17: 0, 10, 30, 50, and 75 wt% water content) were recorded at room temperature on FT-IR spectrometer Nicolet 6700 (Thermo Fisher Scientific, Waltham, MA, USA) by ATR method (without ATR correction).

### 3.2. Synthesis of NADES and ILs

NADES were prepared based on our reported ultrasound-assisted procedure [32]. Two or three-components listed in Table 4 were placed in a glass vial. Then, a calculated amount of deionized water was added. The vial was sealed with a screw-cap and the mixture was sonicated (37 kHz, 30 W) at 40 °C until a homogeneous liquid was formed (10–30 min). In total, 24 different NADESs were obtained and examined. Symbols and compositions (including molar ratios) of NADESs used throughout the study are shown in Table 4.

IL [TEA] [OAc]·AcOH, χ_AcOH_ = 0.75 (mole fraction) was synthesized using a typical procedure: 0.1 mol of triethylamine was placed in a round-bottomed flask equipped with a stirring bar. The flask was placed in an ice bath and stirred vigorously. Then 0.3 mol of acetic acid was added dropwise, and the mixture was stirred for at least 1 h. The resulting mixture was then evaporated using a rotary evaporator at 60 °C, 10^−1^ bar for 6 h (^1^H-NMR (400 MHz, DMSO) δ ppm = 2.81 (q, *J* = 7.3 Hz, 6H), 1.85 (s, 9H), 1.07 (t, *J* = 7.3 Hz, 9H); ^13^C-NMR (101 MHz, DMSO) δ 172.84, 45.11, 21.89, 9.36) [33].

**Figure 6:**
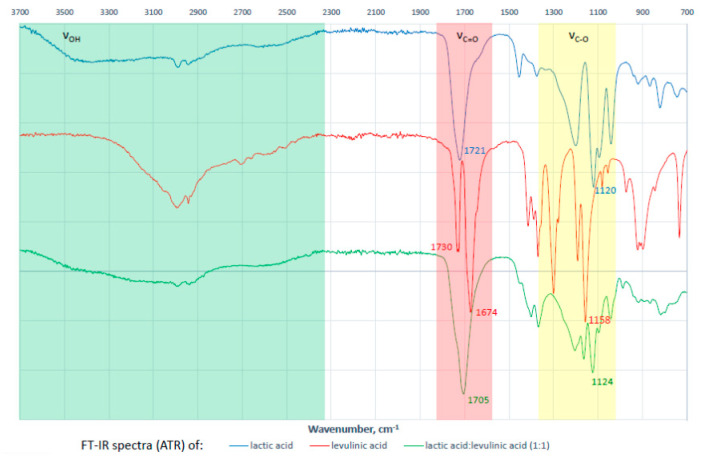
Changes in FT-IR spectra during NADES 17 formation (lactic acid:levulinic acid; 1:1).

The formation of a specific NADES supramolecular structure based on hydrogen bond interactions was observed by IR spectra. Broadening and shifts of characteristic IR vibration bands are perfect evidence/proof of this. For example, distinctive changes were observed in IR spectra for stretching vibrations: νOH, νC=O, νC-O of lactic and levulinic acid forming NADES 17 (Figure 6).

### 3.3. Development of NADES-SLE and IL-SLE Extraction Procedures

#### 3.3.1. Preliminary Evaluation of NADES-SLE Extraction Efficiency

1.5 mL of each extraction solvent (NADES listed in Table 4) was added to dried spinach (100 mg) in a 2.0 mL microtube. Extraction was performed by shaking for 20 min, after which the extracted sample was centrifuged in a microcentrifuge at 6000 rpm for 5 min and the supernatant collected in a new microtube and used for subsequent analyses.

The effects of water content on NADES (10, 20, 30, 50%), sample weight (50, 100, 150 mg), extraction time (10, 20, 40 min) were evaluated using the same extraction and centrifugation procedure described above. All experiments were performed in triplicate.

#### 3.3.2. Preliminary Evaluation of IL-SLE Extraction Efficiency

1 mL of each extraction solvent (ILs listed in Table 4) was added to dried spinach (150 mg) in a 2.0 mL microtube. Extraction was performed by shaking for 30 min, after which the extracted sample was centrifuged in a microcentrifuge at 6000 rpm for 5 min and the supernatant was collected in a new microtube and used for subsequent analyses.

The effects of IL:water ratio (1:0; 1:1; 2:1; 1:2; 4:1; 1:4), sample weight (100, 150, 200, 250, 350 mg), extraction time (10, 20, 30, 60, 120 min), and sample pH (3, 5, 7, and 9) were evaluated using the same extraction and centrifugation procedure as described above. In addition, the effect of sonication on extraction efficiency was investigated. All experiments were performed in triplicate.

Extraction efficiency using ILs and NADES was also compared with water and traditional organic solvents, i.e., acetone, ethanol, methanol, ethyl acetate, and methanol:ethanol:water (1:1:1; *v*/*v*/*v*).

### 3.4. Chromatographic Conditions

Chromatographic analysis of 20-E was carried out using UHPLC-UV system (Merck Hitachi, Darmstadt, Germany) with a model L-2160U binary pump (Merck Hitachi, Darmstadt, Germany), model L-2350U column oven, model L-2200 and model L-2400U UV detector (Merck Hitachi, Darmstadt, Germany). EZ Chrom Elite System Manager software was used for control and data handling. The separation of compounds was carried out a reversed phase (RP) Zorbax 300SB-C18 (150 × 4.6 mm; 2.7 µm) chromatographic column. The mobile phase consisted of acetonitrile (A) and 0.05% trifluoroacetic acid in water (B). The gradient program was as follows: 0–3 min 10% (A), 3–8 min 20% (A), 8.1–10 min 10% (A). The column temperature was maintained at 30 °C and injection volume was 5 µL. The flow rate of the developed method was 1 mL/min. The analyses were carried out using the analytical wavelength λ = 242 nm.

## 4. Conclusions

In this study, a green, simple, novel, and desirable extraction procedure using NADES-based and IL-based combined with UHPLC-UV was developed and validated for extraction and analysis of 20-E in *Spinacia oleracea* L. Among the tested NADES and ILs, lactic acid:levulinic acid mixture (1:1; v:v) and [TEA] [OAc]·AcOH were selected as the most promising solvents. Compared with conventional solvents, NADES gave higher extraction yields than methanol, ethanol, ethyl acetate, acetone, water, and methanol:ethanol:water mixture (1:1:1; *v*/*v*/*v*). Our method displayed favorable linearity, precision, limit of detection, and recovery. The optimized method was applied for the evaluation of real samples for the determination of 20-E at levels between 17.1 and 885 µg/g. The presented method is a promising analytical tool for the determination of ecdysone plant matrices, and possesses many advantages such as simple, rapid, and environmental friendly.

## Figures and Tables

**Table 1:** Analytical characteristics of NADES-SLE-UHPLC-UV and IL-SLE-UHPLC-UV methods.

Parameters		NADES-SLE-UHPLC-UV	IL-SLE-UHPLC-UV
Linear range (µg/g)/R^2^		0.5–30/0.9982	0.5–30/0.9994
LOD/LOQ (µg/g)		0.17/0.5	
Precision RSD (%)	1 µg/g	7.7	6.8
	10 µg/g	2.6	6.2
	25 µg/g	0.4	5.8
Accuracy RE (%)	1 µg/g	9.1	6.2
	10 µg/g	5.2	6.3
	25 µg/g	1.1	4.1
Recovery R (%)	1 µg/g	88.1	86.2
	10 µg/g	90.3	88.5
	25 µg/g	93.4	88.8

**Table 2:** Determination of 20-E in spinach samples using NADES-SLE-UHPLC-UV and IL-SLE-UHPLC-UV methods.

Sample	20-E Content (µg/g Dry Mass)	SD (µg/g Dry Mass)
Fresh spinach 1	878	30
Fresh spinach 2	228	8.8
Fresh spinach 3	65.3	4.7
Fresh spinach 4	55.6	3.7
Fresh spinach 5	101	8.9
Fresh spinach 6	108	9.1
Fresh spinach 7	54.6	5.2
Frozen spinach 1	264	20
Frozen spinach 2	162	15
Frozen spinach 3	50.8	4.6
Frozen spinach 4	17.1	1.4
Frozen spinach 5	41.4	3.9
Frozen spinach 6	48.3	2.7
Frozen spinach 7	32.4	2.2
Spinach leaves 1	656	56
Spinach leaves 2	885	24
Spinach leaves 3	23	1.0
Spinach stalks 1	279	25
Spinach seeds 1	50	3.0
Spinach seeds 2	44	1.9

**Table 3:** Comparison of NADES-SLE-UHPLC-UV and IL-SLE-UHPLC-UV procedures with other reported procedures.

No	Sample Preparation	Extractant	Extraction Time	Analysis	Column	Mobile Phase	Analysis Time/Recovery	Ref.
1	SLE	methanol	48 h	HPLC-MS	Spherisorb 5ODS2 (150 × 4.6 mm, 5 μm)	2-propanol:water (12:88; *v:v*)	11 min/80%	[12]
2	SLE	ethanol	72 h	HPLC-UV	C18 ODS (250 × 4.6 mm, 5 μm)	methanol:water (45:55; *v*:*v*)	-	[28]
3	UAE	ethylene oxide: propylene oxide (1:1; *v*:*v*)	72 h	HPLC-UV	Waters Delta Pak C18 (150 × 3.9 mm)	methanol:water (40:60; *v*:*v*)	11 min/88.7%	[29]
4	UAE	methanol	3 h	HPLC-UV	Kromasil (250 × 4.6 mm)	dichloromethane:2-propanol:water (125:40:3; *v*:*v*:*v*)	60 min	[30]
5	UAE	methanol	3 h	HPLC-UV	Zorbax-TMS (250 × 4.6 mm; 5 µm)	A: acetonitrile:2-propanol (5:2; *v*:*v*) B: water containing 0.1% TFA	60 min	
6	UAE	methanol	3 h	HPLC-UV	Spherisorb 5ODS2 (250 × 4.6 mm; 5 µm)	A: acetonitrile:2-propanol (5:2; *v*:*v*) B: water containing 0.1% TFA	50 min	
7	UAE	methanol	3 h	HPLC-UV	Spherisorb 5ODS2 (250 × 4.6 mm; 5 µm)	A: acetonitrile:2-propanol (5:2; *v*:*v*) B: water containing 0.1% TFA	50 min	
8	UAE	methanol	3 h	HPLC-UV	ACE C18 (150 × 4.6 mm; 5 µm)	methanol:water (45:55; *v*:*v*)	-	
9	SLE	methanol	3 h	HPLC-UV	C18 Luna (250 × 4.6 mm; 5 μm)	11% 2-propanol containing 0.1% TFA	60 min	[31]
10	SLE	NADES levulinic acid: lactic acid (1:1; *v*:*v*)	20 min	UHPLC-UV	Zorbax 300SB-C18 (150 × 4.6 mm; 3.5 μm)	A: acetonitrileB: water containing 0.05% TFA	10 min/93%	This work
11	SLE	IL triethylammonium triacetate	2 h	UHPLC-UV	Poroshell 120 EC-C18 (50 × 3.0 mm; 2.7 µm)	A: acetonitrileB: water containing 0.05% TFA	6 min/88%	This work

**Table 4:** List of NADES and ILs used for extraction

Symbol	Name
**NADESs**	
NADES1	choline chloride:citric acid (1:1)
NADES2	choline chloride:lactic acid (1:1)
NADES3	choline chloride:tartaric acid (1:1)
NADES4	choline chloride:levulinic acid (1:1)
NADES5	acetylcholine chloride:citric acid (1:1)
NADES6	acetylcholine chloride:levulinic acid (1:1)
NADES7	levulinic acid:citric acid (1:1)
NADES8	levulinic acid:malic acid (1:1)
NADES9	acetylcholine chloride:malic acid (1:1)
NADES10	acetylcholine chloride:lactic acid (1:1)
NADES11	levulinic acid:malic acid (1:2)
NADES12	levulinic acid:malic acid (1:4)
NADES13	levulinic acid:malic acid (4:1)
NADES14	levulinic acid:malic acid (2:1)
NADES15	choline chloride:lactic acid (1:2)
NADES16	choline chloride:lactic acid (2:1)
NADES17	lactic acid:levulinic acid (1:1)
NADES18	lactic acid:levulinic acid (2:1)
NADES19	lactic acid:levulinic acid (1:2)
NADES20	levulinic acid:pyruvic acid (1:1)
NADES21	choline chloride:lactic acid: levulinic acid (1:1:1)
NADES22	choline chloride:lactic acid: levulinic acid (1:1:2)
NADES23	choline chloride:lactic acid: levulinic acid (2:1:2)
NADES24	choline chloride:malic acid (1:1)
**ILs**	
[BMIm]AlCl_4_	1-butyl-3-methylimidazolium tetrachloroaluminate
[BMIm]NTf_2_	1-butyl-3-methylimidazolium bis(trifluoromethylsulfonyl)imide
[BMIm]MeSO_4_	1-butyl-3-methylimidazolium methylsulfate
[EMIm]OcSO_4_	1-ethyl-3-methylimidazolium octylsulfate
[EMIm]OTf	1-ethyl-3-methylimidazolium trifluoromethanesulfonate
[EMIm]EtSO_4_	1-ethyl-3-methylimidazolium ethylsulfate
[HMIm]Cl	1-hexyl-3-methylimidazolium chloride
[MOIm]Cl	1-methyl-3-octylimidazolium chloride
[TEA] [OAc]·AcOH	triethylammonium triacetate

## Supplementary Materials

The following are available online.
