# Supplementary material for: Highly Efficient Extraction Procedures Based on Natural Deep Eutectic Solvents or Ionic Liquids for Determination of 20-Hydroxyecdysone in Spinach
Source: Molecules. 2020 Oct 15;25(20):4736. doi: 10.3390/molecules25204736 (PMC7587567; doi:10.3390/molecules25204736)
Supplement: Supplementary file 1 [file molecules-25-04736-s001.pdf]

SUPPLEMENTARY MATERIAL

Highly Efficient Extraction Procedures Based on Natural Deep Eutectic Solvents or Ionic Liquids for Determination of 20-Hydroxyecdysone in Spinach

Sylwia Bajkacz [1,2,*], Kornelia Rusin [1], Anna Wolny [3], Jakub Adamek [2,4], Karol Erfurt [3], and Anna Chrobok [3]

[1] **Department of Inorganic Chemistry, Analytical Chemistry and Electrochemistry,** Faculty of Chemistry, Silesian University of Technology , B. Krzywoustego 6, 44-100 Gliwice, Poland; korneliasliwka@op.pl
[2] Biotechnology Center of Silesian University of Technology, Krzywoustego 8, 44-100 Gliwice, Poland; jakub.adamek@polsl.pl
[3] Department of Organic Chemical Technology and Petrochemistry, Faculty of Chemistry, Silesian University of Technology, Krzywoustego 4, 44-100 Gliwice, Poland; ancziixx@gmail.com (A.W.); karol.erfurt@polsl.pl (K.E.); Anna.Chrobok@polsl.pl (A.C.)
[4] Department of Organic and Bioorganic Chemistry and Biotechnology, Faculty of Chemistry, Silesian University of Technology, Krzywoustego 4, 44-100 Gliwice, Poland
* Correspondence: sylwia.bajkacz@polsl.pl

Academic Editor: Monika Waksmundzka-Hajnos and Miroslaw Hawryl

Received: 23 September 2020; Accepted: 13 October 2020; Published: 15 October 2020

Summary

This supporting information file includes additional results and information as described in the text of the main article including:

Figure S1. Effect of the sample weight (A) and extraction time (B) on 20-E extraction efficiency from spinach using NADES-SLE procedure (NADES, lactic acid: levulinic acid (1:1; v/v)).

Figure S2. Effect of the IL dilution (A), IL pH (B), sample weight (C) and extraction time (D) on 20-E extraction efficiency from spinach using IL-SLE procedure (IL, triethylammonium triacetate).

Figure S3. Comparison IL-SLE and NADES-SLE procedures with another extraction procedures.

Table S1. List of Gutmann donor numbers for anions included in the studied ionic liquids.

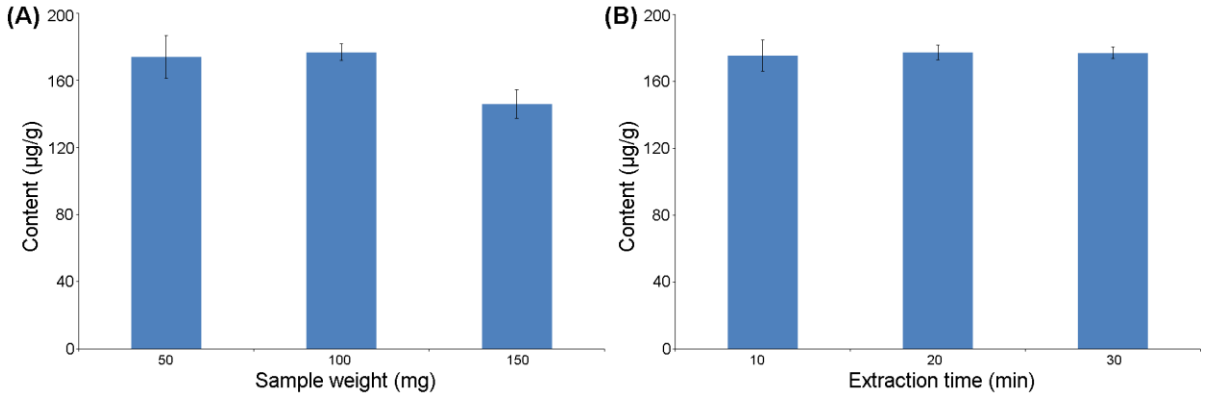

Figure S1. Effect of the sample weight (A) and extraction time (B) on 20-E extraction efficiency from spinach using NADES-SLE procedure (NADES, lactic acid:levulinic acid (1:1; v/v)).

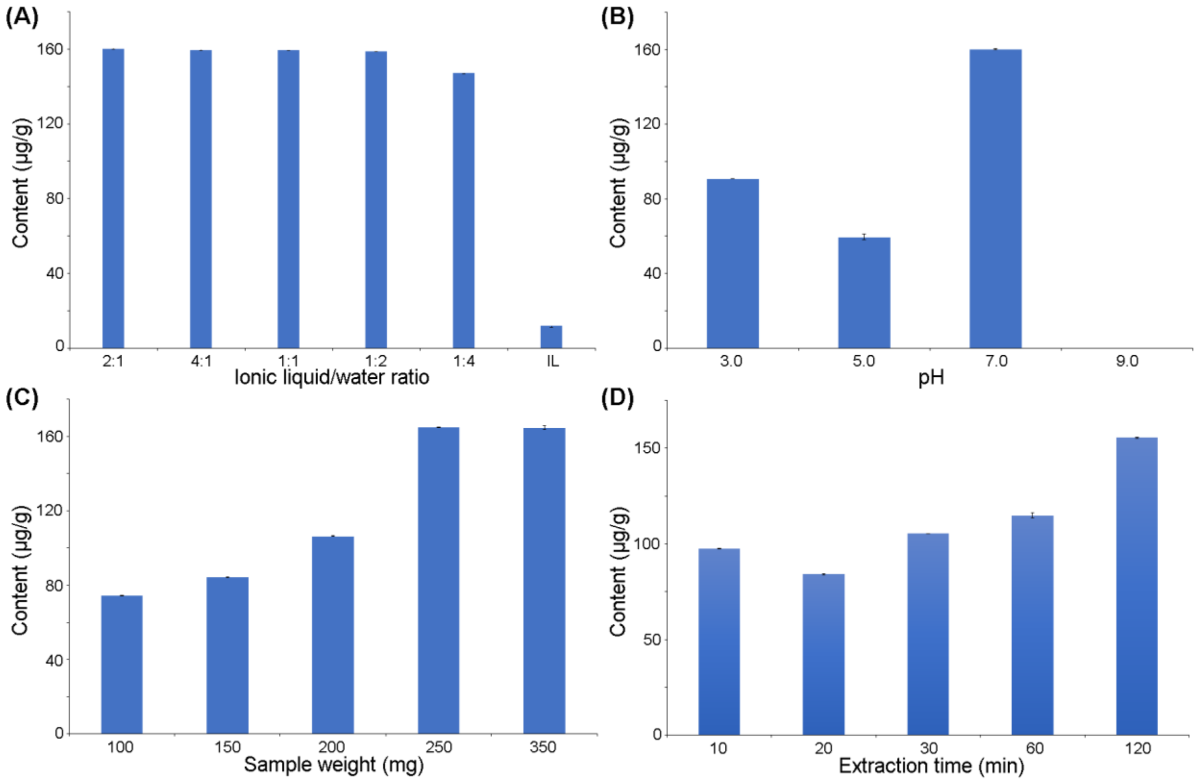

Figure S2. Effect of the IL dilution (A), IL pH (B), sample weight (C) and extraction time (D) on 20-E extraction efficiency from spinach using IL-SLE procedure (IL, triethylammonium triacetate).

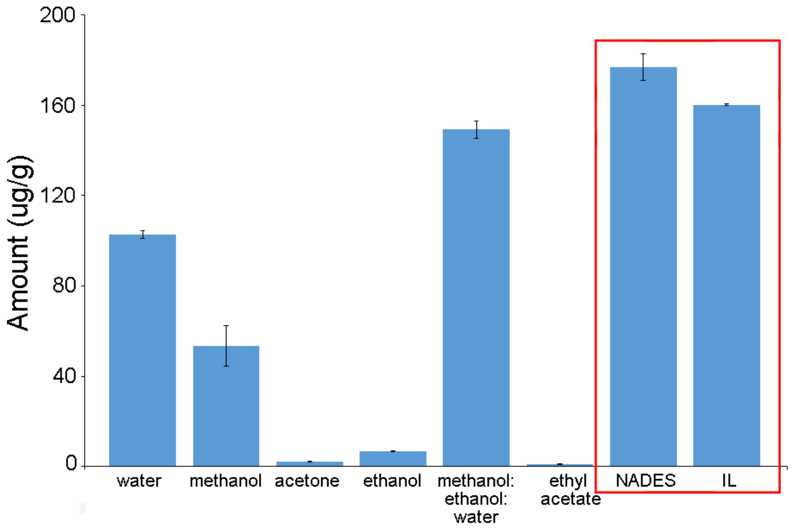

Figure S3. Comparison of NADES-SLE and IL-SLE procedures with conventional extraction solvents.

Table S1. List of Gutmann donor numbers for anions included in the studied ionic liquids.

**Anion of the IL**	**Gutmann donor number (DN)**
NTf_2^-	11.2
OTf^-	20.4
$OcSO_4^-$	20.6
$EtSO_4^-$	22.3
$MeSO_4^-$	–
$AlCl_4^-$	24.3
Cl^-	72.2
$(OAc)_3^-$	126.9
